# Hygiene

**Published:** 1881-06

**Authors:** 


HYGIENE.—"Train up a child in the way he should go, and when he is old he will not depart from it," sayeth the wise man. A decade and more ago we prevailed upon the Commissioners of Public Schools of Baltimore to permit the delivery of lectures, with illustrations, on *Hygiene and Physiology* to the advanced classes in the grammar schools of the city, and for three years lectured five times per week upon those important subjects; and, if the testimony of Superintendent Creery and the most intelligent among the principals of the grammar schools may be taken in evidence, they were of great advantage to both the teachers and pupils in those schools.

It would, doubtless, interest the public, who sustain the schools, to know of the policy that discontinued those instructions but the journal is not the best place for making it known at present. But to return. If early and successful efforts are demanded in the promotion of the public health, they should be primarily impressed upon the young, and then, with their intelligent co-operation, the older members of the community or household can soon be educated up to a proper appreciation of the laws of hygiene. We propose to lay before our readers, from time to time, in as condensed a manner as practicable, such facts and rules concerning health as will prove advantageous to them, and, as occasion favors, add our own personal testimony and experience thereto, without leading them from the useful by glittering or plausible generalities.

